# Visualizing Hydrogen Oxidation Reaction Deactivation on a Polycrystalline Pt Electrode Surface Suppressed by Melamine: A Scanning Electrochemical Microprobe Study

**DOI:** 10.1021/acsami.6c00045

**Published:** 2026-03-24

**Authors:** Masaki Sampei, Daisuke Noda, Kenta Hayashi, Naoto Todoroki, Toshimasa Wadayama

**Affiliations:** Graduate School of Environmental Studies, 13101 Tohoku University, Sendai 980-8579, Japan

**Keywords:** hydrogen oxidation reaction, activity map, platinum, melamine, scanning electrochemical microprobe, electron backscatter diffraction, surface crystallographic orientation

## Abstract

Pt supported on carbon black (Pt/C) is generally used as an anode and cathode catalyst in polymer electrolyte fuel cells. Owing to the highly complex surface microstructure of Pt/C, electrochemical studies employing single-crystal Pt surfaces are powerful but insufficient alone for a comprehensive understanding of the catalytic properties and surface modification effects by organic compounds. This study investigated the dependence of the hydrogen oxidation reaction (HOR) activity of a polycrystalline Pt electrode surface on grain crystallographic orientation and the influence of surface modification by melamine. As a prerequisite, the 2D HOR activity map obtained by a scanning electrochemical microprobe was successfully correlated with the 2D grain crystallographic orientation map determined by electron backscatter diffraction by using indentation marks made by a nanoindenter on the electrode surface as a guide. Low-activity grain regions had orientations close to (100), whereas moderate- and high-activity grain regions exhibited orientations close to (110) and (111), respectively. Then, HOR deactivation was monitored in situ by conducting square-wave potential cycling in a 0.1 M NaClO_4_ + 0.01 M HClO_4_ mixed solution with or without melamine. Changes in the HOR activity map revealed that melamine effectively suppressed HOR deactivation, even under oxidation and reduction conditions of the Pt electrode surface, by stabilizing the microstructures of the (111)- and (110)-oriented surface grains. These findings demonstrate that grain-resolved 2D activity maps provide essential information about the relationship between grain properties and catalytic activity for the design of efficient catalyst surfaces.

## Graphical abstract

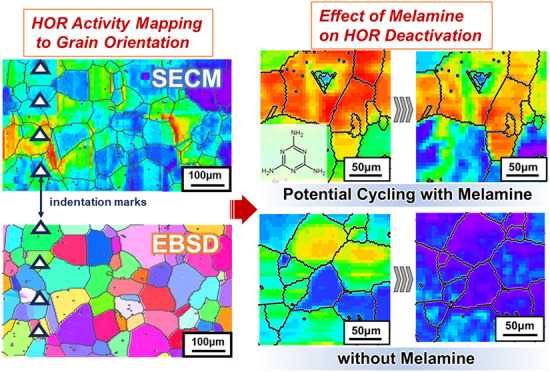

## 1 Introduction

Electrocatalytic reactions on material surfaces, such as metals, alloys, and oxides, are predominantly determined by the atomic arrangement on the surface and electrochemical environment.^1–4^ To develop novel high-efficiency catalysts, an essential strategy is to increase the effective surface area per unit weight, which is typically achieved using catalyst materials such as nanoparticles (NPs). For instance, Pt NPs supported on carbon black (Pt/C) are generally used as cathode and anode catalysts for polymer electrolyte fuel cells (PEFCs).^5,6^ Practical Pt/C catalysts comprise Pt NPs with a wide size distribution on an unstructured carbon black surface containing various defects or other elements.^7–9^ Their initial pristine activity is known to be sensitive to the shape of the NPs at the beginning of life.^10–13^ To clarify the intrinsic catalytic properties of the Pt/C surface, the mechanisms of the oxygen reduction reaction (ORR) on the PEFC cathode and the hydrogen oxidation reaction (HOR) on the PEFC anode were investigated using “well-defined” single-crystal surfaces of Pt [Pt­(*hkl*), *hkl* = 111, 110, 100].^14,15^ The ORR activity was found to be orientation dependent and increased in the order (110) > (111) > (100). Indeed, the ORR activity of octahedron-shaped Pt alloy NPs, where the surface facets are mainly (111) with corners and edges, was higher than that of cube-shaped ones with (100) facets.^16,17^ Thus, the surface characteristics of Pt NPs, specifically the atomic arrangement, size, and shape, are a crucial consideration in synthesizing practical Pt/C catalysts.^18,19^ Designing novel catalyst surfaces requires a comprehensive understanding of the correlations of surface geometrical, electronic, and catalytic properties. While electrochemical studies using single-crystal surfaces of Pt­(*hkl*) can provide deeper insights into the catalytic properties, they are insufficient for understanding practical properties, because of the highly complex nature of the surface microstructures of Pt/C and Pt-M/C (M = Co, Ni, and other transition metals) catalysts. Thus, well-acknowledged factors that affect catalytic properties, including NP size and atomic arrangements on the Pt surface and Pt/C interface, have yet to be elucidated.^19,20^

Surface modification employing organic compounds has been demonstrated to be effective in enhancing the catalytic properties of Pt/C and Pt-M/C.^21–24^ For example, pioneering work by Asahi et al. showed that the introduction of melamine enhanced not only the ORR activity but also the durability by destabilizing surface oxidation agents, such as O_ad_ and/or OH_ad_ species.^25–27^ However, excess melamine on the catalyst surface acts as a surface-poisoning species against the ORR.^28,29^ Thus, considering the complexity of the catalyst surface, even at the beginning of life, and its dynamic changes under the operating conditions of PEFCs,^30^ optimizing the melamine coverage of the Pt (Pt-M) surface is indispensable. However, the optimal amount of melamine that balances the catalytic activity and surface stability remains under investigation.

This study was inspired by a scanning electrochemical microprobe (SECM) 2D mapping report for a polycrystalline Pt electrode (pseudosingle-crystal grains) conducted by Wang and Wipf.^31^ The polycrystalline Pt electrode surface was divided by grain boundaries into micrometer-sized grains with different crystallographic orientations. As schematically shown in Figure 1, the two-dimensional (2D) map of surface grain crystallographic orientation (a) was drawn by electron backscatter diffraction (EBSD). The HOR activity at every surface grain (b) was estimated by SECM, which has been used to clarify the dependence of electrocatalytic properties on surface crystallographic orientation, surface grain size, and coordinatively unsaturated defect sites such as grain boundaries.^31–43^ The resulting 2D HOR activity map was correlated with the surface grain crystallographic orientation by using indentation marks made by a nanoindenter on the electrode surface as a guide (c). After confirming satisfactory correlations of the HOR activity 2D map drawn by SECM and the corresponding surface grain orientations analyzed by EBSD, we extended this experimental platform to visualize the deactivation of the HOR by conducting square-wave potential cycling in a 0.1 M NaClO_4_ + 0.01 M HClO_4_ mixed solution. Furthermore, we demonstrated that this methodology was also effective at clarifying the stabilization effect of the polycrystalline Pt electrode surface by an organic modifier, i.e., melamine. Changes in the 2D HOR activity map indicated that surface modification by an optimal amount of melamine stabilized the microstructures of the surface grains and grain boundaries, especially the (111)- and (110)-oriented grains. Consequently, the pristine HOR activity of the polycrystalline Pt electrode was effectively maintained, even under potential cycling over the range of 1.2–0.6 V vs reversible hydrogen electrode (RHE), which corresponds to the oxidation and reduction cycles of Pt.

**Figure 1:**
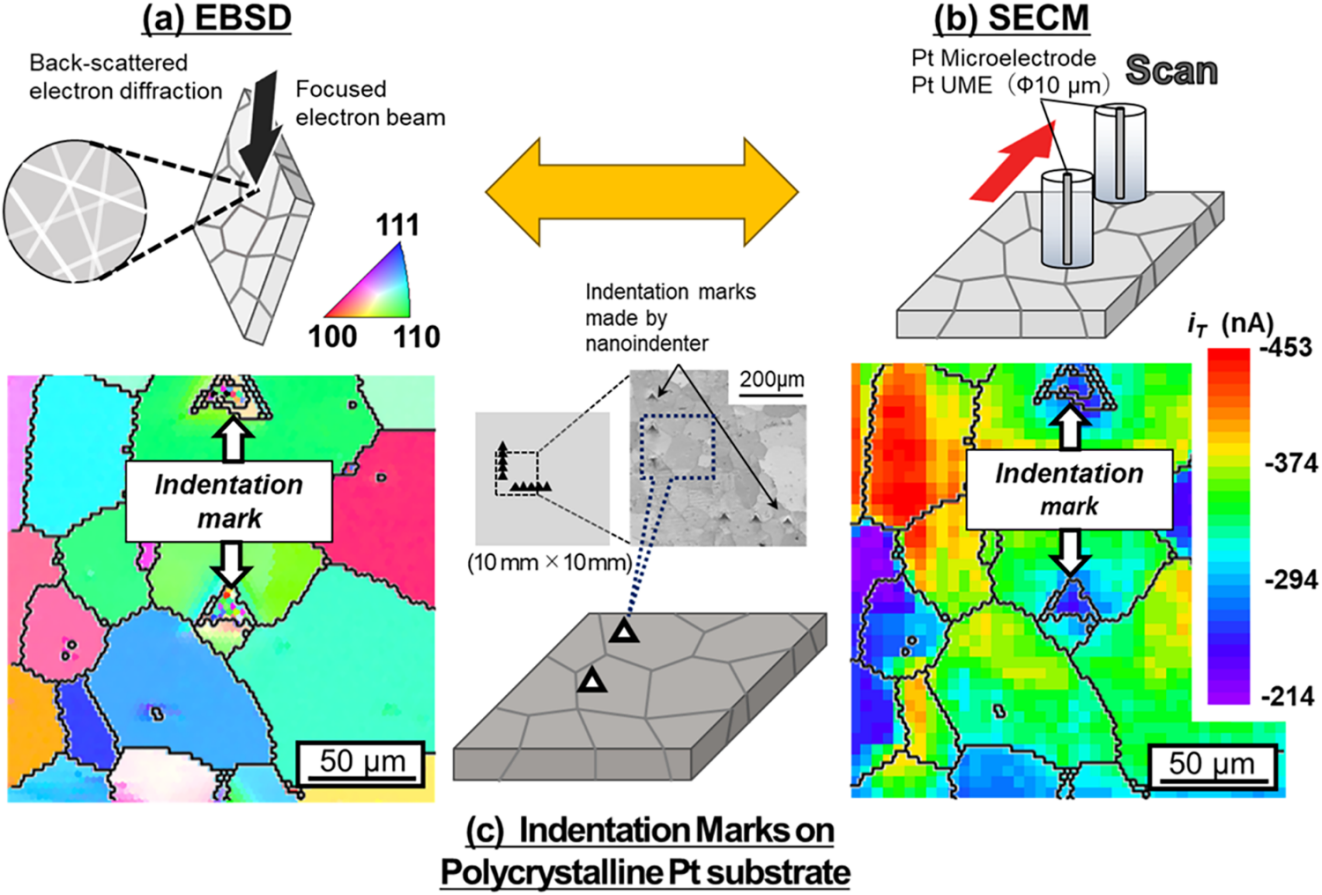
Schematic for correlating the hydrogen oxidation reaction (HOR) activity with the crystallographic orientation of every surface grain using micrometer-sized, triangle-shaped indentation marks made by a nanoindenter on the Pt substrate surface as a guide. Two-dimensional maps of (a) surface grain crystallographic orientation by electron backscatter diffraction (EBSD) and (b) HOR activity by scanning electrochemical microscope (SECM). (c) Indentation marks on the polycrystalline Pt substrate.

## 2 Experimental Methods

### 2.1 Polycrystalline Pt Substrate and Crystallographic Orientations of Surface Grains

A polycrystalline Pt substrate (10 mm × 10 mm, *t* = 1 mm) was polished using Al_2_O_3_ powder (0.3 and 0.05 μm) and successively ultrasonicated in ultrapure water, acetone, and ethanol for 10 min each. Subsequently, the substrate was preannealed at 1000 °C for 15 h under an ultrahigh vacuum (∼10^–7^ Pa) to stabilize the grain sizes and orientations by facilitating grain growth. Thereafter, triangle-shaped indentation marks with a size of 10 μm were made on the preannealed substrate surface using a nanoindenter (TTX-NHT, Anton Paar). The indentation marks served as a guide for correlating the HOR activity with the crystallographic orientation of specific surface grains. The preannealed and marked substrate was cleaned by three repeated cycles of Ar^+^ sputtering and annealing at 1000 °C for 10 min in an ultrahigh vacuum chamber. The crystallographic orientations of the surface grains were analyzed by EBSD using a probe size and an acceleration voltage of 1.5 μm and 20 eV, respectively. In general, the effective depth resolutions of EBSD orientation analysis are generally 20–100 nm, depending upon the accelerating voltage of the incident electron, beam size etc.^44,45^ Surface images were acquired by field-emission scanning electron microscopy (FESEM; Hitachi High-Tech SU-70 scanning electron microscope equipped with an EBSD system). This enabled a 2D map of the crystallographic orientations of the surface grains to be displayed with a density distribution of the orientations in the form of an inverse pole figure (IPF).

### 2.2 Electrochemical (EC) Measurements

For the EC measurements, including SECM, a 0.1 M NaClO_4_ + 0.01 M HClO_4_ mixed solution was prepared using research-grade chemicals (HClO_4_, Kanto Chemical Co.; NaClO_4_, Fujifilm Wako Pure Chemical Corporation) and ultrapure water (Milli-Q, 18.2 MΩ·cm). To modify the polycrystalline Pt electrode surface, water-soluble melamine monomer (99.0 + %; Fujifilm Wako Pure Chemical Corporation) was preadded to the mixed solution at a concentration range of 0.01–1 μM. The counter electrode was a Pt wire (0.05 mm in diameter), and the reference electrode was an Ag/AgCl electrode (saturated KCl, Hokuto Denko). The potential was converted to the RHE reference scale according to the literature.^33,38^ The geometrical surface area of the polycrystalline Pt electrode was determined using an O-ring (0.062 cm^2^; Karlez, DuPont). All EC measurements were conducted at room temperature.

### 2.3 HOR Activity Mapping

The SECM setup (HV-402E, Hokuto Denko) for constructing the 2D HOR activity map is schematically shown in Figure 2(a). The HOR activity was generally estimated in the tip generation/substrate collection (TG/SC) mode.^46^ In this mode, a bipotentiostat independently controls the potentials of a working polycrystalline Pt electrode (*E*_S_) and a Pt ultramicroelectrode probe (Pt UME; Sensolytics 04–00,007; 10 μm in diameter) (*E*_T_). While *E*_S_ was held at a potential at which the HOR can proceed (0.46 V vs RHE), *E*_T_ was held at a potential at which the hydrogen evolution reaction (HER) can proceed (−0.54 V vs RHE). Because H_2_ bubble formation on the Pt UME surface hinders the accurate evaluation of the HOR activity,^39^ a relatively high pH (pH ≈ 2.7) was employed in this study. To visualize the grain-size-dependent HOR activity as a 2D map, the polycrystalline Pt working electrode surface was scanned with a Pt UME placed at a distance of approximately 7 μm. A detailed procedure for the positioning of the Pt UME is described in the Supporting Information (SI). The diameter of the Pt UME governs the spatial resolution of the SECM map^47–49^ and, thus, the resolution of the acquired 2D map is at least >10 μm. As illustrated in Figure 2(b), H_2_ generated by the HER on the Pt UME diffuses to the working electrode surface where it is consumed in the HOR. Its consumption rate depends on the local HOR activity where the Pt UME is positioned. Therefore, through the reaction loop of the HOR and HER, the local HOR activity affects the HER rate on the Pt UME, i.e., |*i*_T_|. In this configuration, the higher the local HOR activity, the higher the |*i*_T_|. Hence, the |*i*_T_| changes measured by the Pt UME should reflect changes in local HOR activity on the working electrode surface. Hereafter, |*i*_T_| is referred to as “HOR activity,” allowing a 2D map of the HOR activity to be visualized based on the |*i*_T_| value at every surface grain. The sampling interval of |*i*_T_| at each position, the scan rate, and the scan area of the Pt UME were fixed at 5 μm, 50 μm/s, and 200 × 200 μm^2^, respectively, based on the values used in the SECM 2D mapping conducted by Wang and Wipf.^31^ The total HOR activity mapping time for the 200 × 200 μm^2^ region was approximately 10 min. By referring to the indentation marks on the working electrode surface, the 2D images of the crystallographic orientations (EBSD map) and HOR activities (SECM map) were correlated to micrometer-order resolution.

**Figure 2:**
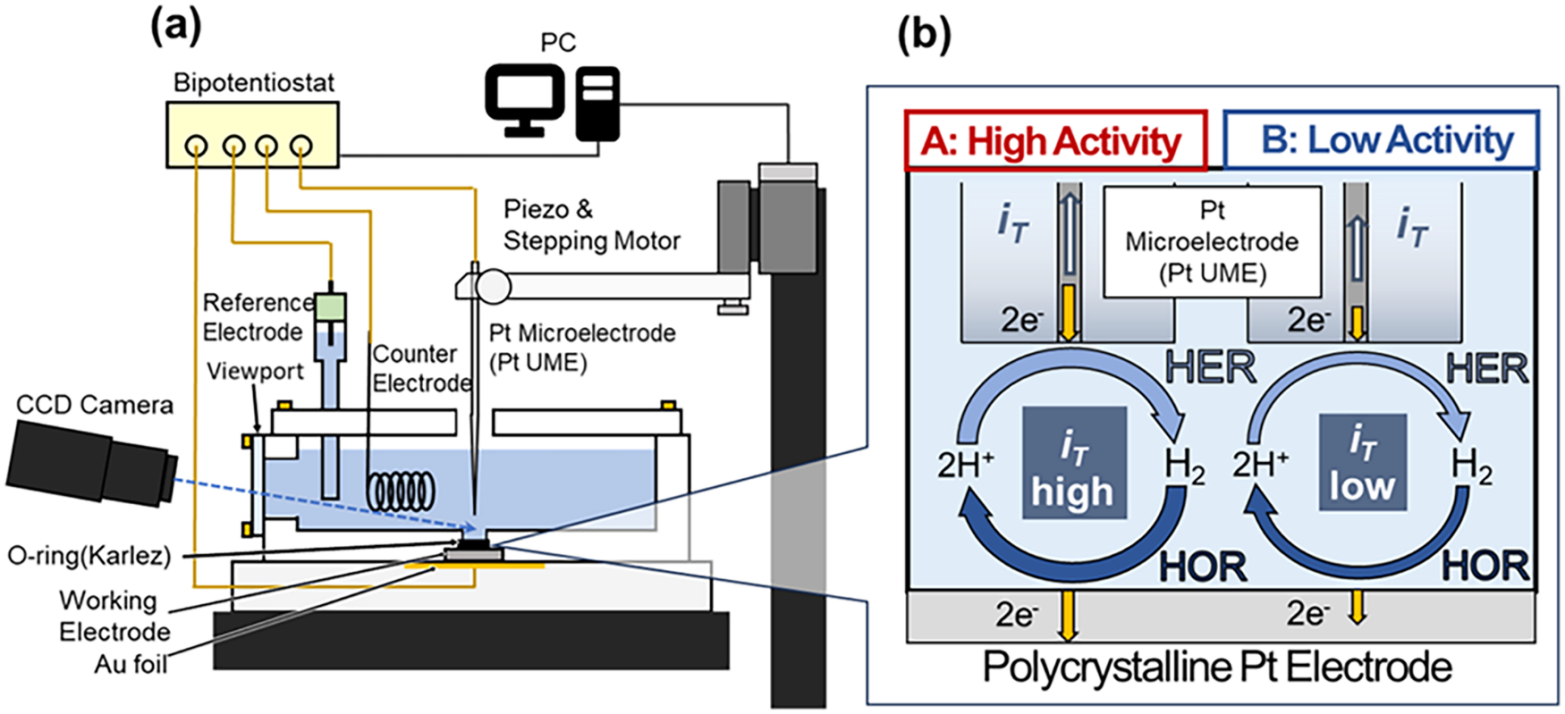
Scanning electrochemical microscopy (SECM) setup for constructing a grain-resolved 2D hydrogen oxidation reaction (HOR) activity map of the polycrystalline Pt electrode surface. (a) Side view of the SECM apparatus and electrochemical cell used in this study. (b) Schematic of HOR activity estimation based on the Pt ultramicroelectrode (UME) current (*i*_T_).

## 3 Results and Discussion

### 3.1 Correlation between HOR Activity and Surface Grain Crystallographic Orientation

Figures 3(a, b) show the 2D maps of the surface grain crystallographic orientation determined by EBSD with an IPF and the HOR activity of the polycrystalline Pt electrode surface acquired by SECM, respectively. The 2D HOR activity map shown in Figure 3 (350 μm × 650 μm) was constructed by overlapping eight pieces of 200 × 200 μm^2^ maps. Each surface grain generally exhibited a specific |*i*_T_| value, depending on the crystallographic orientation. Low-activity grain regions exhibited orientations close to (100), whereas the orientations of the moderate- and high-activity grain regions were close to (110) and (111), respectively [Figure 3(c)]. Thus, an excellent correlation between the crystallographic orientation and HOR activity maps was achieved with micrometer-order resolution.

**Figure 3:**
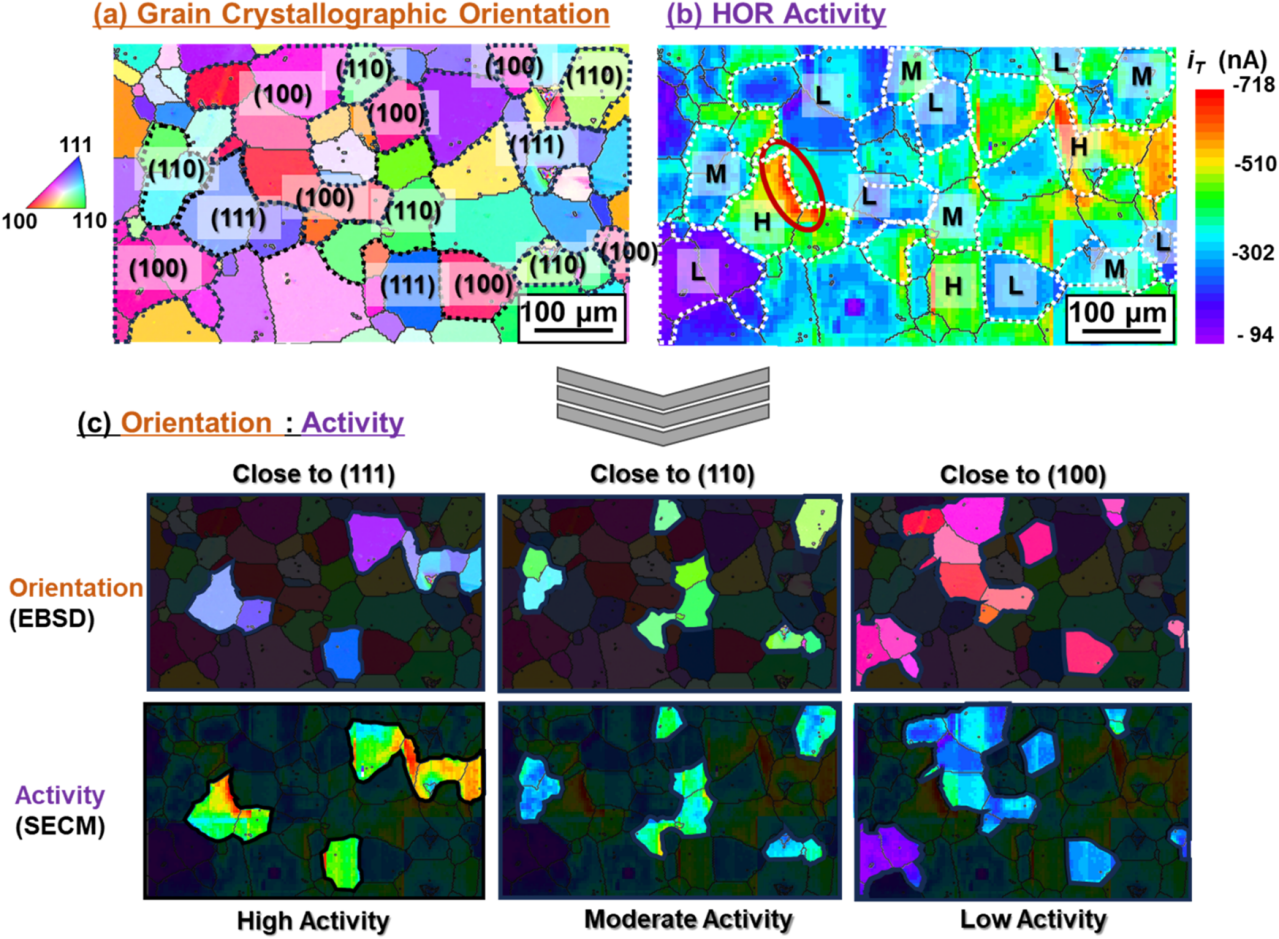
(a) Two-dimensional map of grain crystallographic orientation normal to the polycrystalline Pt working electrode surface determined by electron backscatter diffraction (EBSD). The inverse pole figure color legend is shown on the left-hand side for reference. Typical grains with orientations close to (111), (110), and (100) are indicated by blue, green, and red colors, respectively, and bordered by dotted lines. Grain boundaries are displayed as black solid lines. (b) Two-dimensional hydrogen oxidation reaction (HOR) activity map estimated by scanning electrochemical microscopy (SECM). The HOR activity at each surface grain is distinguished by a color corresponding to an |*i*_T_| value on the right-hand side: high (H; red to yellow), moderate (M; green to sky blue), and low (L; blue to violet). (c) Correlation between the grain orientation (a) and HOR activity (b) for typical grains with orientations close to (111), (110), and (100).

Marković et al.^15^ reported that the exchange current density for HER/HOR follows the order Pt(111) ≪ (100) < (110), by using their developed single-crystal Pt disk electrode (RDPt­(*hkl*)­E) method. By contrast, the HOR activity trend estimated in this study shows the following order: (111)- > (110)- > (100)-oriented grains. Indeed, Wang and Wipf reported a similar trend for the orientation-dependence of the HOR activity on a polycrystalline Pt electrode (pseudosingle-crystal grains) in a 10 mM H_2_SO_4_ + 0.1 M K_2_SO_4_ solution using the SECM method.^31^ It is well-known that the reaction rate of the HOR (and HER) on Pt surfaces is quite fast and, thus, quite low mass transport resistance must be achieved to estimate the accurate rate constant of the HOR. Durst et al.^50^ pointed out that RDE-based method tends to underestimate HOR/HER activity due to the limitation of mass transport. Thus, the difference in the HOR activity estimation methods might cause the experimentally determined HOR activity trend. In addition, the electrochemical potential of the polycrystalline Pt electrode (*E*_S_ = 0.46 V vs RHE in this work) during drawing the SECM maps might be influenced for the estimated HOR activities. Although the primary adsorption reaction at *E*_S_ = 0.46 V is hydrogen adsorption, OH species might also adsorb on (100) and (110)-oriented grains,^51^ deactivating the HOR through active site blocking by OH_ads_. The influence of Cl^–^ contamination cannot be excluded for the HOR activity estimation^52^ because an Ag/AgCl electrode is used as a reference electrode of the SECM cell. However, considering the HOR activity trend for the pseudosingle-crystal grains,^31^ where mercury/mercurous sulfate reference electrode is used as a reference electrode, the Cl^–^ contamination of the electrolyte seems to have little impact on the estimated HOR activity trend in this study. Furthermore, the HOR activity map (b) is overlaid with 8 pieces of 200 × 200 μm^2^ maps, and the total acquisition time is ca. 10 × 8 = 80 min. Nevertheless, the activity trend of (111) > (110) > (100) is maintained, irrespective of each area of the 200 × 200 μm^2^ maps. The results suggest that the stability of the electrochemical measurements is enough for the following discussions, revealing satisfactory correlation between the HOR activity and corresponding grain crystallographic orientation. Although, at present, we cannot explicitly explain the different trends for the orientation-dependent HOR activities, the SECM method is preferable to judge the HOR activity order for the polycrystalline Pt working electrode surface.

The HOR involves the following possible elementary reaction steps: dissociation of H_2_ molecules into adsorbed hydrogen atoms (H_ads_) (Tafel step), dissociation of H_2_ into one adsorbed hydrogen atom and one proton (Heyrovsky step), and charge transfer (Volmer step).^40^ Two possible HOR mechanisms, namely, the Tafel–Volmer and Heyrovsky–Volmer mechanisms, have been proposed to date. Although the HOR mechanism is still under debate, the consensus is that H_ads_ is a key intermediate species in the reaction.^53,54^ In the case of on-top site adsorption, H_ads_ binds more weakly on Pt(111) than on Pt(100),^41^ leading to a higher HOR activity on Pt(111) than on Pt(100). Close inspection of the 2D HOR activity map showed changes in the vicinity of specific grain boundaries, even within the same surface grain [for example, the region marked by a red circle in Figure 3(b)]. An SEM image of the polycrystalline Pt electrode surface and a cross-sectional high-angle annular dark-field scanning transmission electron microscopy (HADDF-STEM) image of the surrounding area of the grain boundary between (997) and (25 15 1) (Figure S1) clearly showed a height difference of less than 1 μm between neighboring grains. In other words, surface grains are not atomically flat with the same orientation but locally change with the curvature, especially around the vicinity of grain boundaries. Similar specific microstructures around the boundaries can be seen in the SEM images of high-index surface grains [Figure S2­(d)]. Such surface microstructures with curvatures might stem from strain release of differently oriented neighboring grains. In this study, the Pt UME with a diameter of 10 μm was placed at a fixed distance of approximately 7 μm from the polycrystalline Pt working electrode surface. Although the influence of this height difference (ca. 1 μm) on the HOR activity cannot be ruled out, the relation between the *i*_T_-value and the distance between the Pt UME and the sample substrate surface (Figure S3 in the SI) suggests that the influence is limited. In addition, the substrate current (*i*_S_) information shown in the SI (Figure S4) also supports that the HOR activity rather than the topography should dictate the |*i*_T_|-mapping. Thus, changes in |*i*_T_| within the same surface grain in the pristine state can be attributed to local crystallographic orientations within the grain or local surface strain. The latter factor, local strain, can be visualized by a Kernel Average Misorientation (KAM) map of EBSD.^55,56^ However, the KAM map cannot always be correlated to the local HOR activity estimated by SECM, as shown in Figure S5 in the SI. Thus, the influence of strain on HOR activity is implied to be limited. Rather than strain, the |*i*_T_|-fluctuation within the grain can be associated with a wide variety of local crystallographic orientations, particularly around the boundaries, compared with those in the center region, resulting in the altered chemical bonding between H_ads_ and Pt atoms. Therefore, specific regions around the grain boundaries exhibit different HOR activities, even within the same grain.

### 3.2 HOR Deactivation Induced by Potential Cycling

In Section 3.1, the HOR activities are successfully correlated with the surface orientations of the grains that are analyzed by EBSD. Surface degradation of the Pt electrode under the practical PEFC operating conditions is one of the keys for understanding the catalytic durability of the Pt and Pt-M alloy catalysts. Therefore, in the Section 3.2, degradation behaviors of the surface-grain-dependent HOR activities are discussed by applying potential fluctuations that simulate PEFC operating conditions. Figure 4 shows the changes in the SECM map induced by 400 cycles of two combinations of upper and lower potentials of square waves [(b)-(i) 1.2 V (3 *s*)–0.6 V (3 *s*) and (b)-(ii) 0.6 V (3 *s*)–0.05 V (3 s) vs RHE] for two independent 200 × 200 μm^2^ regions of the polycrystalline Pt electrode surface. The former potential range (b)-(i) corresponds to the oxidation and reduction cycles of the surface Pt atoms, accompanied by their dissolution.^57–61^ The latter, (b)-(ii), mainly corresponds to hydrogen underpotential deposition on Pt atoms, where Pt dissolution should be moderate. Consequently, surface degradation at every surface grain can be discussed through a comparison of the results by conducting two potential cycling conditions. As shown in Figure 4, the square-wave potential cycling (b)-(i) caused a decrease in |*i*_T_|, irrespective of the pristine values (before potential cycling). Lopes et al. investigated the surface microstructural change of clean Pt(111) induced by a step–hold–step protocol between 0.05 and 1.15 V vs RHE in 0.1 M HClO_4,_ ^60^ in which the protocol resulted in a high density of ad-islands and structurally degraded steps, accompanied by the dissolution of Pt in the electrolyte. Indeed, SEM images collected before and after potential cycling (Figure S2) showed that this potential cycling condition introduced submicrometer-order surface roughness and defects to the electrode surface. As discussed in Section 3.1, the SECM-estimated HOR activity is sensitive to the local surface structures. Thus, the marked decrease in |*i*_T_|, i.e., HOR deactivation, can be explained by microstructural surface degradation, such as coordinatively unsaturated Pt sites, generated by the introduction of roughness and defects by the potential cycling of (b)-(i). Irreversibly formed Pt oxides or contaminating inclusions possibly located near the surface vicinity might also have decreased the HOR activity of the specific grains. Notably, less HOR-active (smaller |*i*_T_|) regions were conspicuous around grain boundaries after potential cycling. SEM images collected before and after the potential cycling clearly showed remarkable changes in the microstructures around the boundaries of high-index grains [Figure S2­(d)]. Surface defects of the substrate, such as grain boundaries and inclusions, are known to act as starting points of corrosion in various electrochemical environments.^62–65^ Probably, such coordinatively unsaturated surface sites should trigger microstructural surface degradation, accompanied by HOR deactivation.

**Figure 4:**
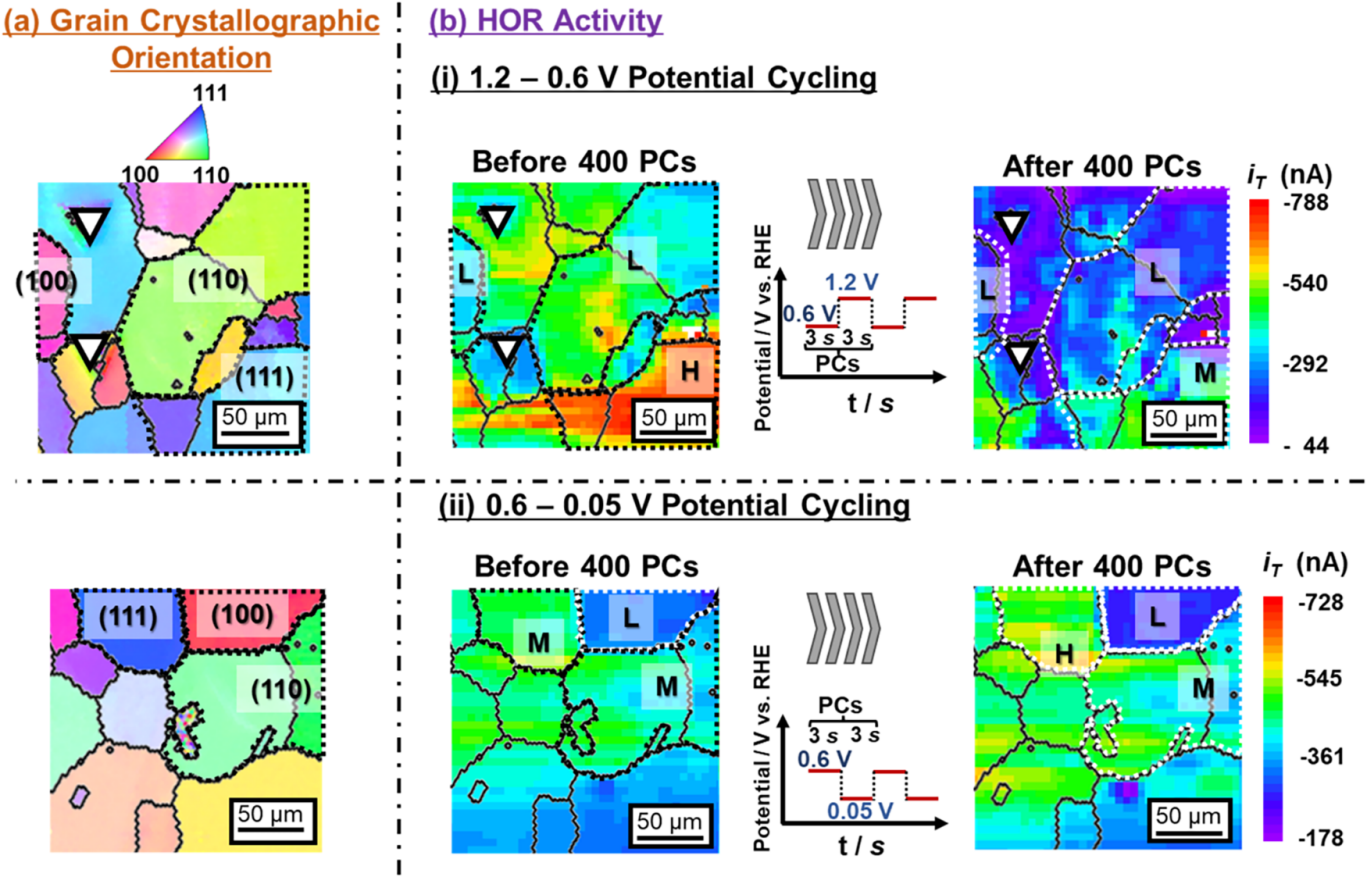
(a) 2D surface grain crystallographic orientation maps for the two independent regions with the inverse pole figure color legend presented on top. (b) The corresponding two-dimensional hydrogen oxidation reaction (HOR) activity maps of the polycrystalline Pt working electrode surface acquired before and after applying 400 square-wave potential cycles (PCs) of (b)-(i) 1.2 V (3 s)–0.6 V (3 s) and (b)-(ii) 0.6 V (3 s)–0.05 V (3 s). Two reference micrometer-sized, triangle-shaped indentation marks are shown in each image of top panels.

By contrast, the 2D HOR activity map remained nearly unchanged under the potential cycling at a range of 0.6 V (3 *s*)–0.05 V (3 *s*) [Figure 4(b)-(ii)]. In this potential range, the possible redox reactions on the Pt electrode surface can be attributed mainly to hydrogen adsorption/desorption. Therefore, unlike the Pt dissolution triggered by oxidation and reduction cycles of the surface Pt atoms during the 1.2 V (3 *s*)–0.6 V (3 *s*) potential cycling [Figure 4(b)-(i)], the surface morphology of the pristine polycrystalline Pt electrode seems to be stable, almost keeping the pristine HOR activities even after 400 cycles.

### 3.3 Dependence of Melamine Concentration on HOR Activity

In the previous Section 3.2, the degradation behaviors of HOR activities of the Pt electrode surface are shown by conducting two different square-wave potential cycling conditions [1.2 V (3 s)–0.6 V (3 s) and 0.6 V (3 s)–0.05 V (3 s)]. As described in Section 1, Introduction, the surface modification of Pt by melamine monomer is known to be effective not only for ORR activity enhancement but also for the surface stabilization of Pt/C and Pt-M/C catalysts. Indeed, the enhancement of the ORR activity of Pt NPs by melamine has been reported, in which the enhancement depends on the melamine concentration in the electrolyte.^25–27^ On the other hand, the effect of melamine addition on the HOR has not been reported to date. Therefore, in this section, the SECM maps for the polycrystalline Pt electrode with different concentrations of melamine are presented and discussed.

Figure 5(b) shows the changes in the 2D HOR activity map upon the addition of 0.01, 0.1, and 1 μM melamine to the 0.1 M NaClO_4_ + 0.01 M HClO_4_ mixed solution and the corresponding grain crystallographic orientation map (Figure 5­(a)). A slight addition of melamine (0.01 μM) resulted in a decrease in |*i*_T_| for grains whose crystallographic orientations are close to (100). This decrease became clearer upon increasing the concentration to 0.1 μM, whereas the (111)-oriented grains maintained almost their pristine HOR activity. Addition of 1 μM melamine caused HOR deactivation on every surface grain. These results suggest that the degree of deactivation is sensitive to the crystallographic orientation of the surface grain. As noted above, (100)-oriented grains were more deactivated than (111)-oriented ones with the addition of 0.1 μM melamine. We have previously reported the melamine-concentration-dependence of the ORR activity and durability of a Pt/high-entropy alloy/Pt­(*hkl*) single-crystal lattice stacking surface, whose surfaces were comprised of almost pure Pt­(*hkl*) lattices.^29^ The ORR activity of Pt/high-entropy alloy/Pt(111) peaked at a melamine concentration of 0.1 μM. By contrast, increasing the concentration from 0.001 to 10 μM resulted in a monotonic decrease in the ORR activity of Pt/high-entropy alloy/Pt(110). Although, to the best of our knowledge, detailed ORR enhancement mechanisms by surface melamine are still under debate, an optimal amount of surface melamine can destabilize adsorbed OH species on the Pt surface, enhancing the ORR through the modifying surface water solvation structure.^30^ These previous reports suggest that melamine surface coverages and molecular orientations for each surface atomic arrangement of Pt govern the ORR activity enhancement: an excess concentration of melamine should inhibit the reaction through surface poisoning, deactivating the ORR. In this study, only a smaller molecular size of adsorbed species (H_ads_) must be considered for the HOR, in contrast to the larger species (O_ads_, OH_ads_ and/or H_2_O_ads_) and/or surface water structure involved in the ORR. Such differences in reaction mechanisms might affect the melamine-concentration-dependence of the activity; hence, the optimum concentrations for the ORR and HOR would be different. Based on the present results (Figure 5), a melamine concentration of at least 1 μM seems to be excessive, deactivating the HOR regardless of the surface grain orientation.

**Figure 5:**
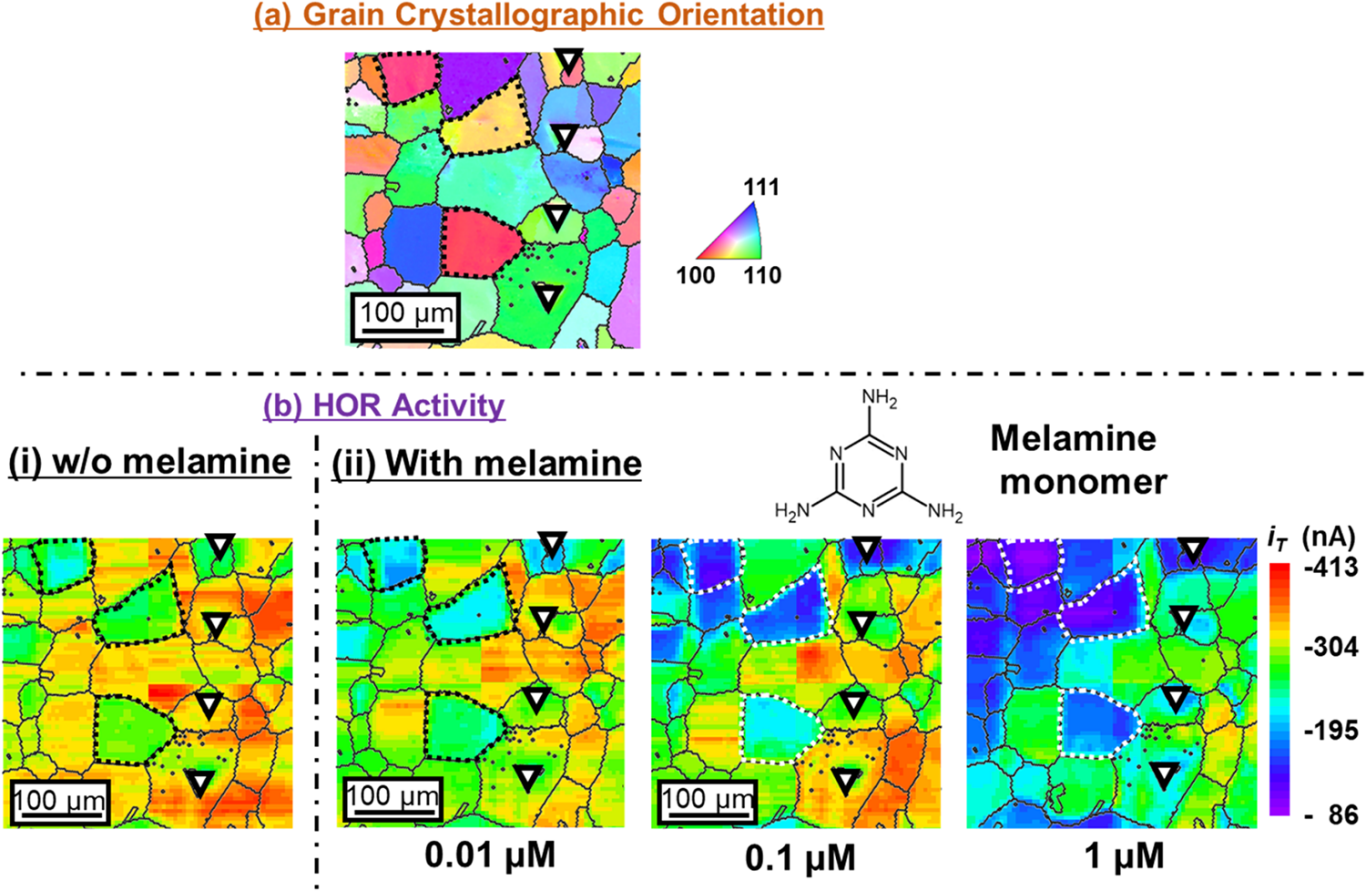
Two-dimensional maps of the (a) surface grain crystallographic orientation analyzed by electron backscatter diffraction (EBSD) with the inverse pole figure color legend on the right-hand side and (b) hydrogen oxidation reaction (HOR) activity maps acquired in the 0.1 M NaClO_4_ + 0.01 M HClO_4_ mixed solution [(i) w/o melamine] and containing 0.01, 0.1, or 1 μM melamine [(ii) with melamine] with four reference micrometer-sized, triangle-shaped indentation marks shown.

### 3.4 Suppression of Surface Degradation by Melamine

Considering the correspondence of the HOR activities and surface grain orientations, the surface stabilization effect by melamine for the polycrystalline Pt electrode is considered in this section. As shown in Figure 5(b)-(ii), the pristine HOR activity of the surface grain with an orientation close to (111) remained nearly unchanged up to the addition of 0.1 μM melamine. Thus, we investigated the effect of adding 0.1 μM melamine on the HOR activity under the potential cycling of 1.2 V (3 s)–0.6 V (3 s), at which severe surface degradation of the Pt electrode occurred [Figure 4(b)-(i)]. The results are listed in Figure 6. Compared with the case without melamine, a remarkable durability enhancement of specific grains was clearly observed upon the addition of melamine [Figure 6(b)]. Except for the region around the indentation mark, at which the distance between the Pt UME and the working electrode surface might change steeply, the decrease in |*i*_T_| was considerably suppressed by melamine addition. This was confirmed by SEM images of the surface acquired after potential cycling (Figure S2). Therefore, melamine modification of the polycrystalline Pt electrode surface effectively preserves HOR activity, even during the oxidation and reduction cycles of surface Pt atoms, particularly those located in the grains with crystallographic orientations close to (111) and (110). On the contrary, the HOR activity decreased significantly in the (100)-oriented grains. Katagiri et al. investigated the molecular orientations of adsorbed melamine on the Pt­(*hkl*) single-crystal surface in 0.1 M HClO_4_ by infrared reflection–absorption spectroscopy.^66^ The results showed that melamine was adsorbed via one amino group on Pt (111) and Pt (110) and two amino groups on Pt(100). Melamine adsorption via two bulky amino groups on (100)-oriented grains appeared to be an obstacle for the HOR. Although no further consideration was made in this study, this difference in melamine orientation on Pt­(*hkl*) might explain the effect of melamine addition, especially on the (111)- and (110)-oriented surface grains.

**Figure 6:**
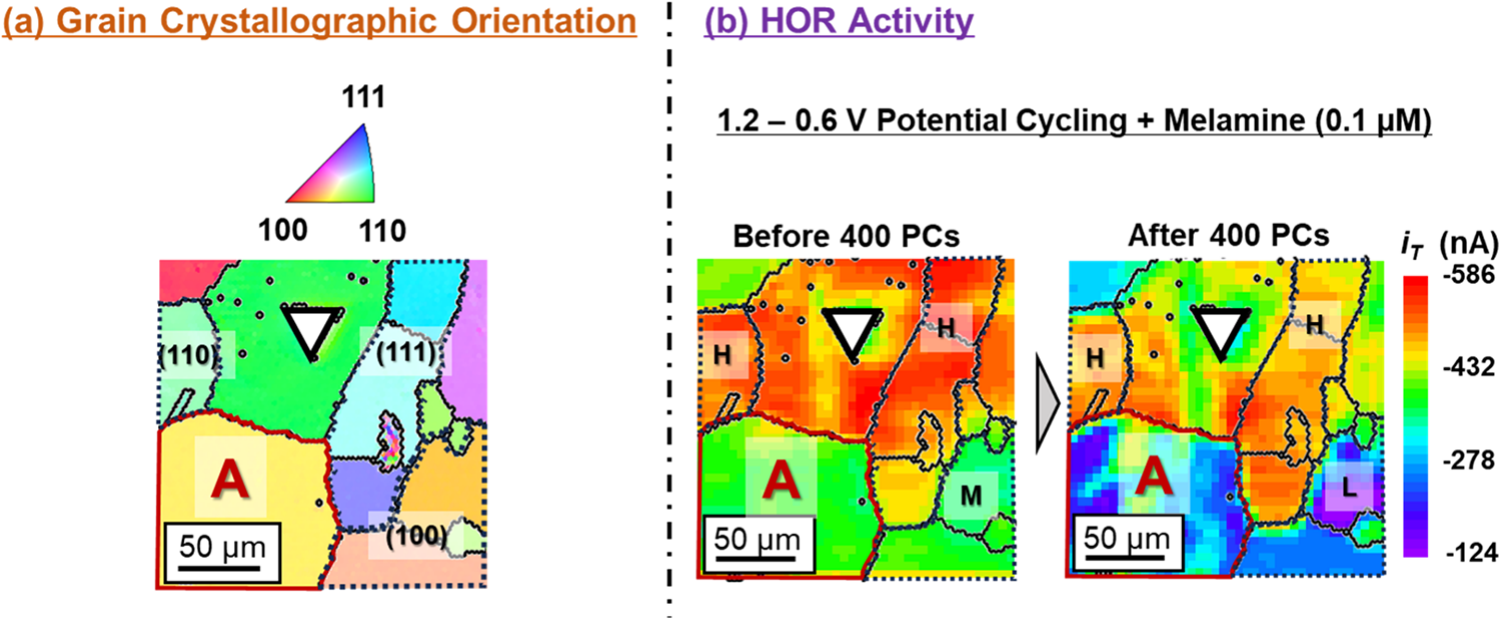
(a) Two-dimensional surface grain crystallographic orientation map. The inverse pole figure color legend is shown on the left-hand side for reference. (b) Two-dimensional hydrogen oxidation reaction (HOR) activity maps acquired before and after applying 400 square-wave potential cycles (PCs) of 1.2 V (3 s)–0.6 V (3 s) in the 0.1 M NaClO_4_ + 0.01 M HClO_4_ mixed solution containing 0.1 μM melamine. The activity level of each grain [high (H; red to yellow), moderate (M; green to sky blue), and low (L; blue to violet)] is specified. One reference micrometer-sized, triangle-shaped indentation mark is shown in each image. Typical grains with orientations close to (111), (110), and (100) and corresponding activity regions are surrounded by dotted lines.

Close inspection of grain A [surrounded by red lines in Figure 6(b)] shows that the relatively uniform |*i*_T_| value became nonuniform upon potential cycling. Nonuniform HOR activity in specific grains also appeared on the SECM maps in Figures 3 and 4. As shown in Figure S2, square-wave potential cycling at 1.2 V (3 *s*)–0.6 V (3 *s*) in the melamine-free mixed solution introduced roughness and defects on the Pt electrode surface, with pronounced surface degradation at the boundaries of high-index grains. On the other hand, the addition of 0.1 μM melamine effectively suppressed surface roughness (right-hand-side SEM images in Figure S2). Furthermore, as mentioned in Section 3.1, nonuniform local microstructures would lead to different HOR activities, even within the same surface grain. Because the orientation of grain A corresponds to that of a high-index grain between low-index (100) and (110) yellow color in Figure 6(a), 400 square-wave potential cycles of 1.2 V (3 s)–0.6 V (3 s) would induce local surface degradation different from that in adjacent regions, leading to nonuniform HOR activity [Figure 6(b)]. We adapted the Pt UME having 10 μm in diameter for detecting |*i*_T_| values, and thereby, special resolution of the HOR 2D maps is estimated to be at least >10 μm. Therefore, when adjacent degraded regions greater than 10 μm in size are formed by the square-wave potential cyclying, local HOR activities can be distinguished even within the same grain. Melamine adsorption is known to be sensitive to surface atomic arrangements.^23,66,67^ The higher affinity of melamine with regions of specific surface atomic arrangements would stabilize the higher affinity regions during conducting 1.2 V (3 s)–0.6 V (3 s) potential cycling, perhaps inducing changes in local HOR activity as appeared in grain “A”. These results reveal that the addition of 0.1 μM melamine effectively stabilizes the surface microstructures of the polycrystalline Pt electrode, even under the oxidation and reduction conditions of Pt. Particularly, the surface melamine adsorbed on (111)- and (110)-oriented grains retains their pristine HOR activities.

## 4 Conclusion

The HOR activities of micrometer-sized surface grains with different crystallographic orientations on a polycrystalline Pt electrode surface in a 0.1 M NaClO_4_ + 0.01 M HClO_4_ mixed solution were mapped in situ by SECM. By using indentation marks made by a nanoindenter on the electrode surface as a guide, we successfully correlated the resulting 2D map with that of the crystallographic orientations of the surface grains analyzed by EBSD. Subsequently, HOR deactivation on the electrode surface under square-wave potential cycling in the mixed solution, with and without melamine, was monitored in situ. Based on the changes in the 2D HOR activity map and the SEM and STEM images, the effect of surface modification by melamine was analyzed. Comparison of the SECM maps after two different potential cycling protocols [1.2 V (3 s)–0.6 V (3 s) and 0.6 V (3 s)–0.05 V (3 *s*)] indicated that HOR deactivation is triggered by the oxidation and reduction cycles of surface Pt atoms occurring at every surface grain and grain boundary. Furthermore, addition of 0.1 μM melamine effectively suppressed HOR deactivation, even under square-wave potential cycling at 1.2 V (3 s)–0.6 V (3 s), probably owing to the stabilization of the surface microstructures of the grains and grain boundaries, particularly the (111)- and (110)-oriented grains. This study demonstrates that grain-resolved 2D HOR activity maps provide essential information about the surface degradation of the polycrystalline Pt electrode and its suppression by melamine.

## Supplementary Material



## Notes

SEM and cross-sectional HADDF-STEM images of differently oriented polycrystalline Pt grains around grain boundaries, SEM images acquired before and after potential cycling at 1.2–0.6 V vs RHE in the mixed solution with and without 0.1 μM melamine, a cyclic voltammetry (CV) curve of the Pt UME that positioned at *d* > 300 μm from the polycrystalline Pt electrode surface, an approach curve of the Pt UME for the polycrystalline Pt electrode surface, and surface strain of the polycrystalline Pt electrode visualized by a Kernel Average Misorientation (KAM) map (PDF)

## †

ISHIFUKU Metal Industry Co., Ltd., Aoyagi 2–12–30, Soka 340–0002, Japan

## ‡

HIROSE ELECTRICc CO., LTD., 2–6–3 Nakagawa Chuoh, Tsuzuki-ku, Yokohama 224–8540, Japan

## §

TOYOTA CENTRAL R&D LABS., INC., Yokomichi 41–1, Nagakute 480–1192, Japan
