# Supplementary material for: Visualizing Hydrogen Oxidation Reaction Deactivation on a Polycrystalline Pt Electrode Surface Suppressed by Melamine: A Scanning Electrochemical Microprobe Study
Source: ACS Appl Mater Interfaces. 2026 Mar 24;18(13):19079–88. doi: 10.1021/acsami.6c00045 (PMC13067234; doi:10.1021/acsami.6c00045)
Supplement: Supplementary file 1 [file am6c00045_si_001.pdf]

Supporting Information

Visualizing Hydrogen Oxidation Reaction Deactivation on Polycrystalline Pt Electrode Surface Suppressed by Melamine: A Scanning Electrochemical Microprobe Study

*Masaki Sampei [a †], Daisuke Noda [a ‡], Kenta Hayashi [a §], Naoto Todoroki [a], and Toshimasa Wadayama [a]**

[a] Graduate School of Environmental Studies, Tohoku University, Sendai 980-8579, Japan

*Email: toshimasa.wadayama.b1@tohoku.ac.jp

Present addresses:

† M.S.: ISHIFUKU Metal Industry Co., Ltd., Aoyagi 2-12-30, Soka 340-0002, Japan

‡ D.N.: HIROSE ELECTRIC CO., LTD., 2-6-3 Nakagawa Chuoh, Tsuzuki-ku, Yokohama 224-8540, Japan

§ K.H.: TOYOTA CENTRAL R&D LABS., INC., Yokomichi 41-1, Nagakute 480-1192, Japan

KEYWORDS: hydrogen oxidation reaction; activity map; platinum; melamine; scanning electrochemical microprobe; electron backscatter diffraction; surface crystallographic orientation

1. Microscopic images of the polycrystalline Pt substrate surface

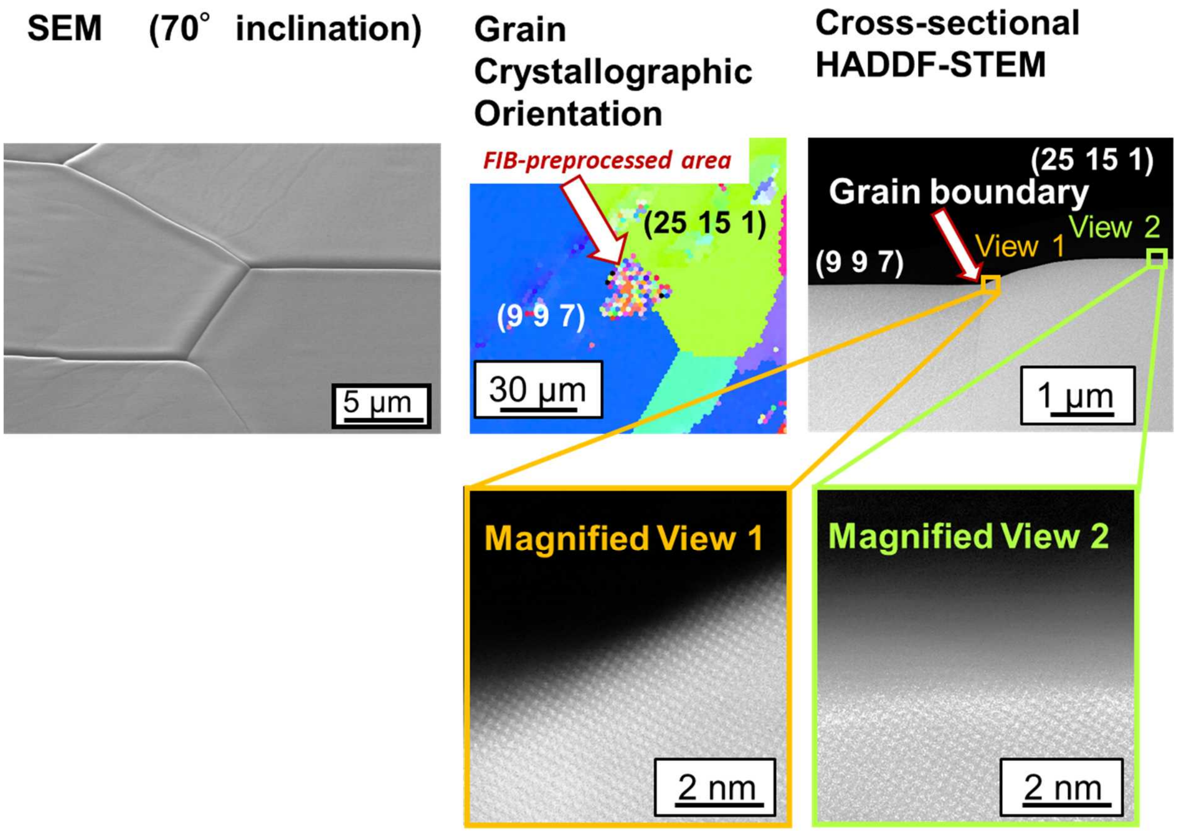

Figure S1. SEM image, crystallo-graphic orientation map, and cross-sectional HADDF-STEM image of the surrounding area of the grain boundary between (997) and (25 15 1) on the polycrystalline Pt substrate surface. Atomically resolved HADDF-STEM images of views 1 and 2 are presented at the bottom.

Figure S1 shows microscopic images of the polycrystalline Pt substrate surface subjected to electrochemical measurements, acquired by field-emission scanning electron microscopy (FESEM; Hitachi High-Tech SU-70 scanning electron microscope equipped with an electron backscatter diffraction (EBSD) system) and cross-sectional high-angle annular dark-field scanning transmission (HAADF-STEM; JEOL JEM-ARM200F) microscopes, respectively. The STEM

samples were pre-processed using a focused ion beam (FIB). As the figure shows, the grains had a height difference in the order of a hundred nanometers, especially around the grain boundaries. Such height difference was also observed for the grain boundary region between the high-index grains (see Figure S2d).

2. SEM images of grains on the polycrystalline Pt electrode surface

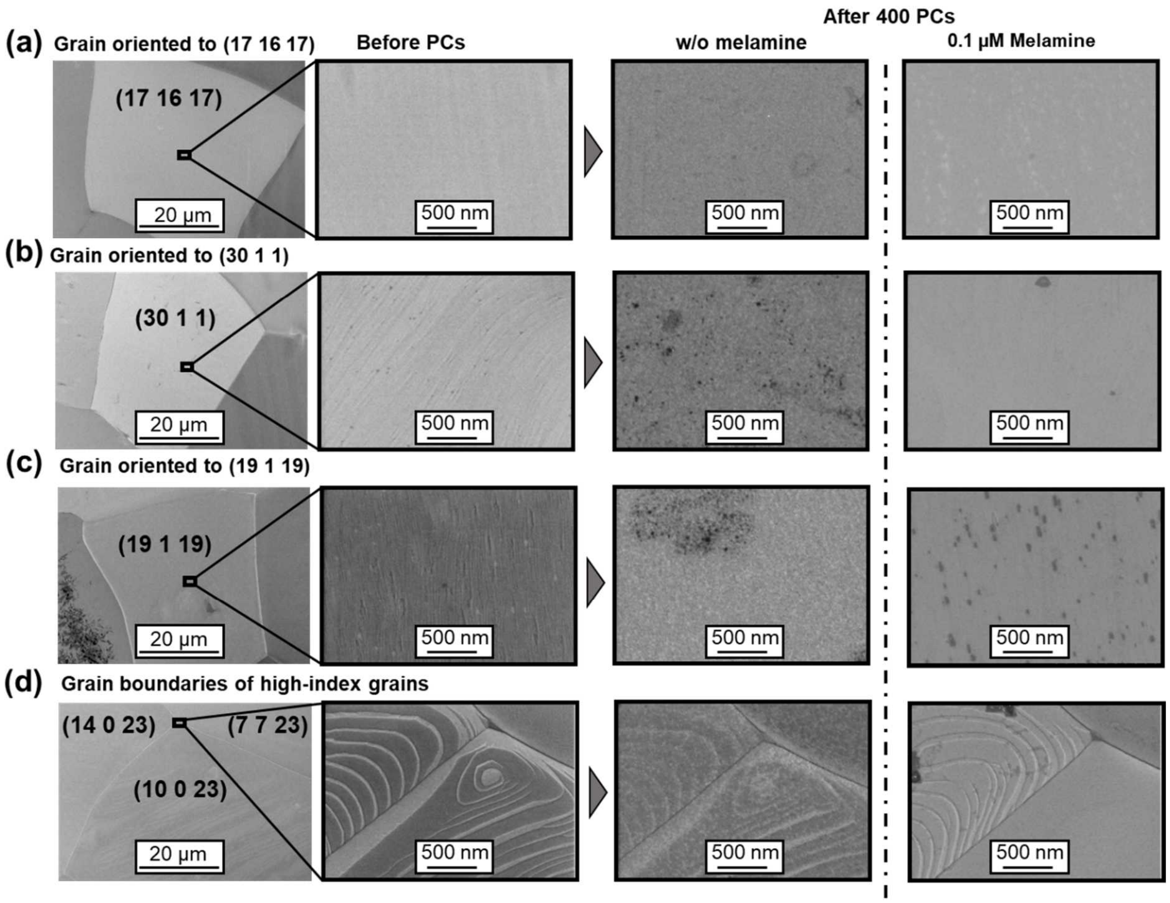

Figure S2. SEM images of surface grains with different crystallo-graphic orientations on the polycrystalline Pt electrode, collected before and after 400 potential cycles (PCs) at 1.2–0.6 V vs. RHE. Low-magnification images of differently oriented surface grains, collected before potential cycling, are presented on the left-hand side. The middle panels show high-magnification images

of the grains before and after 400 PCs. The SEM images taken after 400 PCs with and without melamine in the 0.1 M $NaClO_4$ + 0.01 M $HClO_4$ mixed solution are shown on the right-hand side. The surface contamination shown in panels (b)–(d) probably caused insufficient surface cleaning during ultrahigh vacuum preparation (before potential cycling) or residual electrolytes (after potential cycling).

Figure S2 shows SEM images of grains with different crystallographic orientations on the polycrystalline Pt electrode surface [(17 16 17) (a); (30 1 1) (b); (19 1 19) (c); and neighboring regions of the (14 0 23)-, (7 7 23)-, and (10 0 23)-oriented grains)] analyzed by EBSD.[1] Regardless of the grain crystallo-graphic orientation, square-wave potential cycling at 1.2–0.6 V vs. RHE introduced surface roughness with some contamination. The addition of 0.1 μM melamine to the 0.1 M $NaClO_4$ + 0.01 M $HClO_4$ mixed solution appeared to keep the surface smooth compared with that observed in the melamine-free solution.

3. Positioning the Pt UME from the working polycrystalline Pt electrode

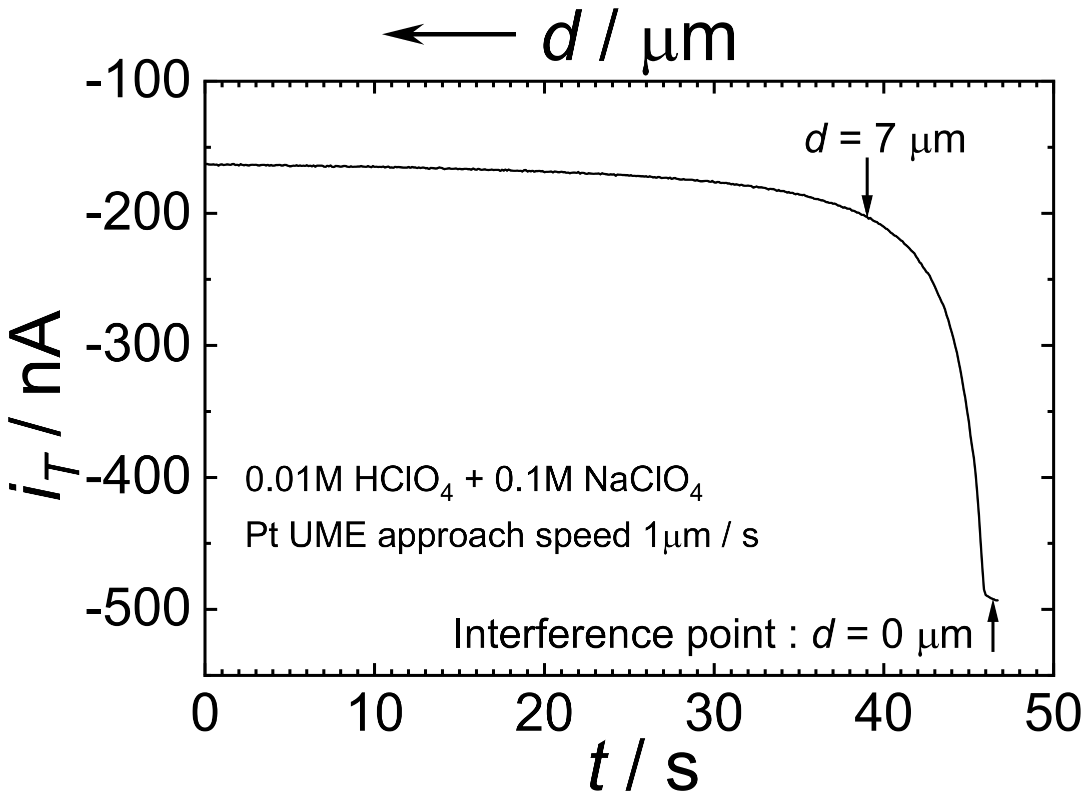

Figure S3. An approach curve of the Pt UME for the polycrystalline Pt electrode surface.

To present a typical dependence of i_T against *d* (a distance between the Pt UME and polycrystalline Pt electrode surface), so called "approach curve", recorded before drawing HOR activity 2D map, is presented in Figure S3. As shown, the increasing $|i_T|$-value with decreasing *d*-value is a typical shape of the approach curve with HOR-active electrode surfaces, like Pt. However, in the actual 2D mapping of HOR activity, it is difficult to determine the exact *d*-value. Instead, we approached the Pt UME to the polycrystalline Pt electrode surface at fixed speed (1 μm/s). This is why Figure S4 has t-axis (bottom x-axis), which indicates how long the Pt UME moved toward the substrate

surface. Around t > 45 (s), interference of the approach curve is apparent Such interference reveals the mechanical contact between the sealing glass of the Pt UME and the working electrode surfaces. Based on this interference point appeared on the approach curve, we can define the point of $d = 0$, as shown in the figure. As we mentioned in experimental section 2.3, the HOR activity 2D maps were collected with d = ca. 7 μm. We retracted the Pt UME for 7 s at the speed of 1 μm / s from the point of $d = 0$, positioning the Pt UME at ca. 7 μm from the electrode surface. As shown in the approach curve, around the $d = 7$ μm ± 1 μm, i_T values can be estimated to be −197 ~ −211 nA. Compared to the fluctuation of the i_T values (ca. 15 nA by the fluctuation of $\Delta d = \pm 1$ μm), the HOR activity 2D maps displayed in this study showed clear contrast of i_T value typically larger than 100 nA or more. Therefore, the HOR activity maps in this study visualize the grain orientation dependence on HOR activity rather than microstructural fluctuation of the polycrystalline Pt electrode surface, particularly around the grain boundaries.

4. Information of substrate current (i_S)

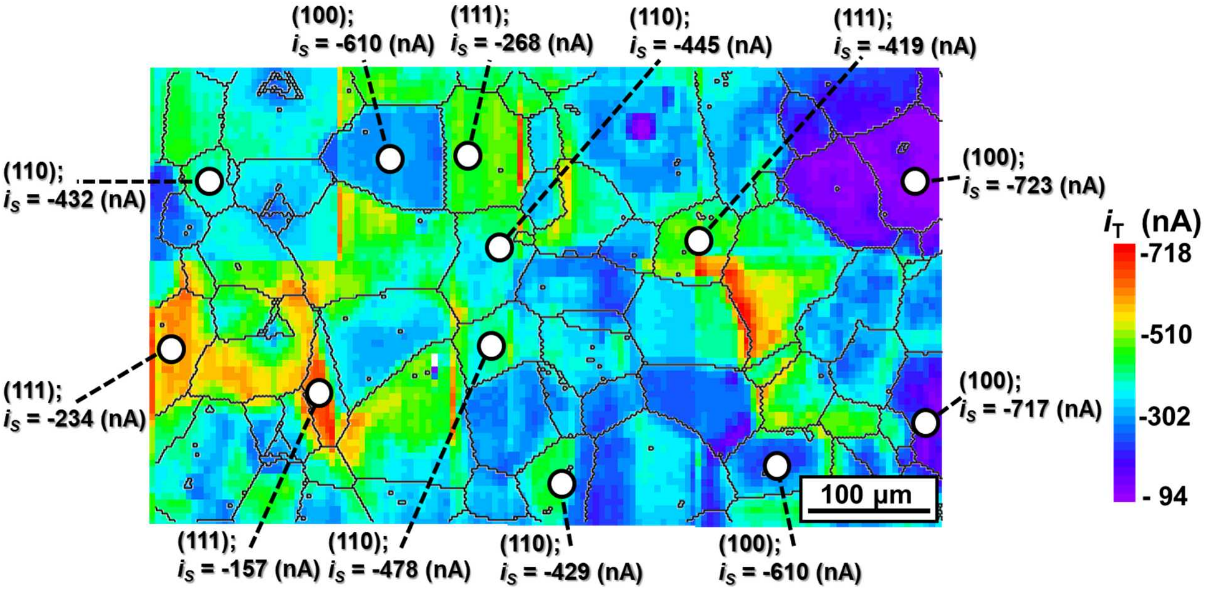

Figure S4. Values of substrate currents (i_S) at the selected points in the HOR mapping

Information of i_S-values (working electrode current) for the several selected points in the HOR activity 2D map is shown in Figure S4. The map used in this figure is identical to that of Figure 3 (b) in the main manuscript. The i_S-values were simultaneously recorded with acquiring the $|i_T|$-map. The negative values of i_S indicate that cathodic reaction is dominant on the polycrystalline Pt electrode surface. This is because ORR can proceed on the Pt substrate surfaces and, thereby, i_S-value represents averaged information of the whole Pt electrode surfaces. Note that it was guaranteed by the previous paper[2] that involvement of ORR had little influence on the HOR activity evaluation in TG/SC mode of SECM; thus, purging solutions by inert gas was unnecessary. Figure S4 clearly showed that the points with larger $|i_T|$-values (i.e. high HOR activity) correspond to smaller negative i_S-values. Because HOR, an anodic reaction, is locally induced on the Pt

substrate surface, at which the Pt UME is positioned, correspondence of the $|i_T|$ with i_S values can be explained by the reaction loop of Figure 2(b) in the main manuscript. The result shown in Figure S4 supports that $|i_T|$-mapping in this study well reflects the HOR activity rather than surface topography.

5. Surface strain of the polycrystalline Pt electrode visualized by KAM map

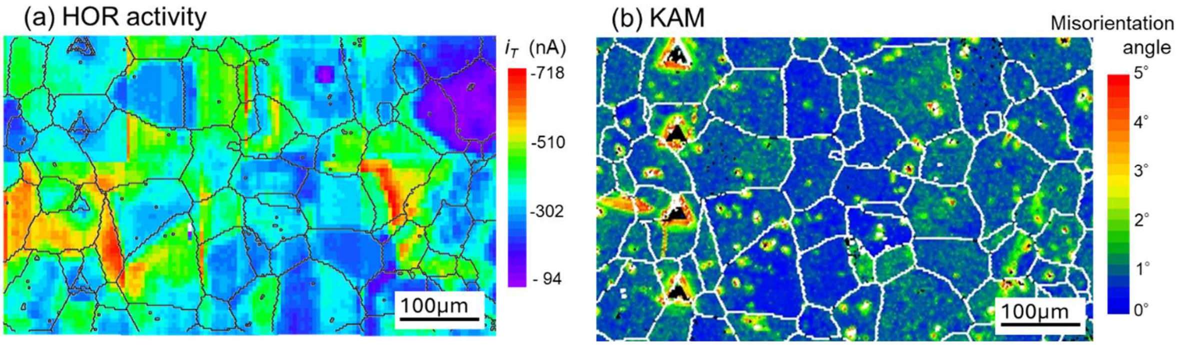

Figure S5. Correspondence of (a) HOR activity and (b)surface strain maps visualized by KAM (Kernel Average Misorientation) method of EBSD for the polycrystalline Pt electrode.

Kernel Average Misorientation (KAM) map of EBSD analysis is a guide of local lattice distortion inside the grains depicted through average misorientation between each neighboring pixel, reflecting strain accumulation and deformation in the microstructure.[3,4] As shown in Figure S5, except of the regions of four triangle-shaped indentation marks, HOR activities judged by SECM and corresponding strains analyzed by KAM are not always corresponded locally. This implies that the influence of the strain on the HOR activity might be small in this study.

6. Cyclic voltammetry curve of the Pt UME

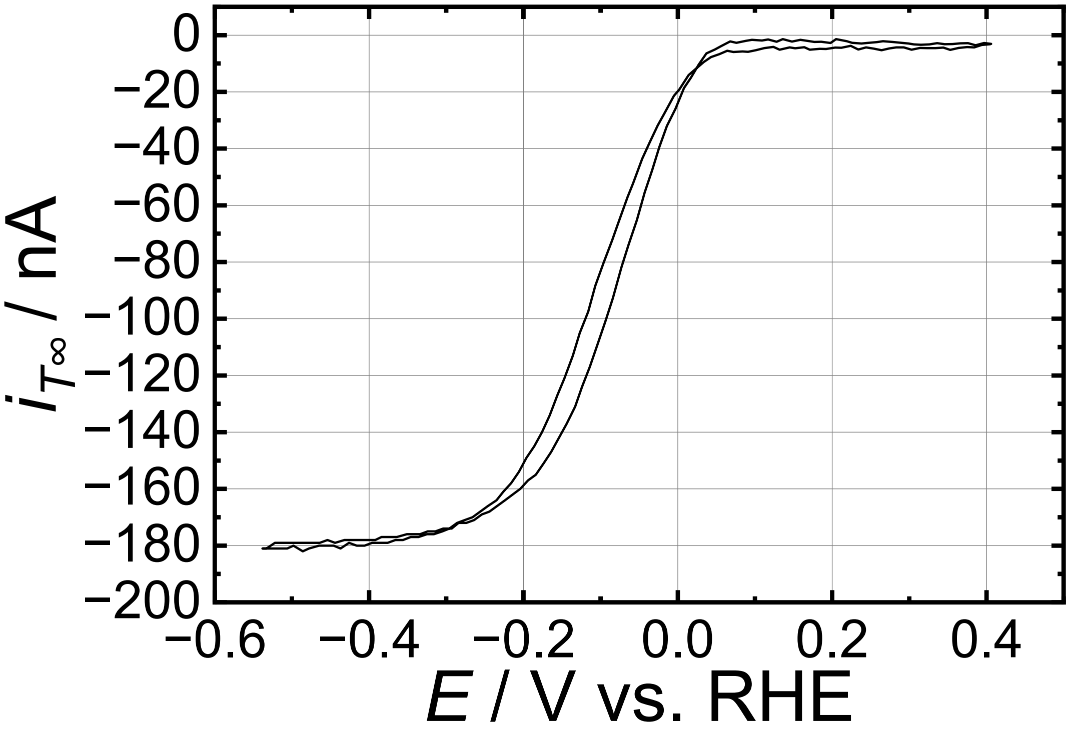

Figure S6. A cyclic voltammetry (CV) curve of the Pt UME that positioned at d > 300 μm from the polycrystalline Pt working electrode surface.

A cyclic voltammetry curve for the Pt UME recorded before the HOR activity 2D mapping is presented in Figure S6. We routinely measured $i_{T,\infty}$-value (the limiting HER current value of which corresponds to $i_{T,}$ value measured at the Pt UME position d > 300 μm; d is a distance between the Pt UME and polycrystalline Pt electrode surface) before the drawing HOR 2D activity maps to check the Pt UME condition. The typical value of $i_{T,\infty}$ was approximately −180 nA (at E_T = −0.54 V vs. RHE). This check also corresponds to “a blank experiment” to confirm that the i_T-value

increase due to the reaction loops shown in Figure 2 cannot be detected under such the Pt UME position ($d > 300$ μm).

References

(1) Hayashi, K.; Kameoka, S.; Tsai, A.-P. NO + CO reaction on polycrystalline palladium foils with specific surface crystallographic orientation: A new approach to develop foil catalysts based on texture control. *Materials Transactions* **2021**, *62* (8), 1089–1096. DOI: 10.2320/matertrans.MT-M2021050.

(2) Zhou, J.; Zu, Y.; Bard, A. J. Scanning electrochemical microscopy: Part 39. The proton/hydrogen mediator system and its application to the study of the electrocatalysis of hydrogen oxidation. *Journal of Electroanalytical Chemistry* **2000**, *491* (1–2), 22–29. DOI: 10.1016/S0022-0728(00)00100-5.

(3) Shin, W-S.; Lee, T.; Sohn, H.; Kim, Y-J.; Park, C. Microstructural and mechanical properties degradation of the acceleration/deceleration zones in stainless steel 316L fabricated by selective laser melting. *Journal of Materials Research and Technology* **2025**, 35, 2215–2225. DOI: 10.1016/j.jmrt.2025.01.183.

(4) Chaudhuri, A.; Sarkar, A.; Suwas, S. Investigation of stress-strain response, microstructure and texture of hot deformed pure molybdenum. *International Journal of Refractory Metals & Hard Materials* **2018**, 73, 168–182. DOI: 10.1016/j.ijrmhm.2018.02.011.
